# Free three-dimensional image software in local extension assessment of oral squamous cell carcinoma: a pilot study

**DOI:** 10.1016/j.bjorl.2022.07.001

**Published:** 2022-07-29

**Authors:** João Pedro Perez Gomes, André Luiz Ferreira Costa, Carlos Takahiro Chone, Albina Messias de Almeida Milani Altemani, João Maurício Carrasco Altemani, Carmen Silvia Passos Lima

**Affiliations:** a Department of Anesthesiology, Oncology and Radiology, Faculdade de Ciências Médicas da Universidade Estadual de Campinas, Campinas, SP, Brazil; b Post-Graduate Program in Dentistry, Universidade Cruzeiro do Sul, São Paulo, SP, Brazil; c Department of Ophthalmology and Otorhinolaryngology, Faculdade de Ciências Médicas da Universidade Estadual de Campinas, Campinas, SP, Brazil; d Department of Pathology, Faculdade de Ciências Médicas da Universidade Estadual de Campinas, Campinas, SP, Brazil

**Keywords:** Oral squamous cell carcinoma, Tumor dimension, Multiplanar reconstruction image, Three-dimensional segmentation image, Positive surgical margin

## Abstract

**Objective:**

Oral Squamous Cell Carcinoma (OSCC) is conventionally treated by surgical resection, and positive surgical margins strongly increase local recurrence and decrease survival. This study aimed to evaluate whether a Three-Dimensional Segmentation (3DS) image of OSCC confers advantage over Multiplanar Reconstruction (MPR) of OSCC using images of computed tomography scan in surgical planning of tumor resection.

**Methods:**

Twenty-six patients with locally advanced OSCC had tumor morphology and dimensions evaluated by MPR images, 3DS images, and Surgical Pathology Specimen (SPS) analyses (gold standard). OSCC resection was performed with curative intent using only MPR images.

**Results:**

OSCC morphology was more accurately assessed by 3DS than by MPR images. Similar OSCC volumes and dimensions were obtained when MPR images, 3DS images and SPS measurements were considered. Nevertheless, there was a strong correlation between the OSCC longest axis measured by 3DS and SPS analyses (ICC = 0.82; 95% CI 0.59‒0.92), whereas only a moderate correlation was observed between the longest axis of OSCC measured by MPR images and SPS analyses (ICC = 0.51; 95% CI 0.09‒0.78). Taking only SPS with positive margins into account, MPR images and 3DS images underestimated the tumor’s longest axis in eight out of 11 (72.7%) and 5 out of the 11 (45.5%) cases, respectively.

**Conclusion:**

Our data present preliminary evidence that 3DS model represents a useful tool for surgical planning of OSCC resection, but confirmation in a larger cohort of patients is required.

**Level of evidence:**

Laboratory study.

## Introduction

Oral Squamous Cell Carcinoma (OSCC) is a malignant epithelial tumor that represents 95% of all forms of Head and Neck squamous Cell Carcinoma (HNSCC).^1^ It is well known that tumor resection with surgical clear margins strongly influences both local recurrence and survival of OSCC patients, or triggers the need for an additional surgery or adjuvant therapy.^2^, ^3^, ^4^, ^5^, ^6^, ^7^ Despite advances in chemoradiation and molecular targeted therapy, the outcome of HNSCC patients has not significantly improved over the past 20 years,^8^, ^9^, ^10^, ^11^ and these findings highlight the importance of complete tumor resection in the treatment of patients.

Locally advanced OSCC may develop in regions close to critical structures, and it is essential to precisely establish the tumor boundaries in order to obtain clear surgical margins and to preserve patient's function and quality of life.^12^

Current clinical practice relies on the use of pre-operative planning of surgical team based on multiplanar reconstruction of HNSCC by computed tomography scan images (MPR), visual appearance and palpation of the tumor and intraoperative frozen sections to guide the extent of resection, followed by the traditional histopathologic analysis of surgical margins performed only in the postoperative period.^12^ Nevertheless, MPR images may not be sufficient to show tumor limits,^13^ and the use of intraoperative frozen sections to identify residual tumor at surgical margins has also been of controversial value.^12^, ^14^, ^15^

In recent years, new technologies have emerged as attempts of facilitating tumor resection with free margins. Near-Infrared (NIR) light spectrum^16^ and NIR fluorescence-guided optical imaging,^12^, ^17^, ^18^, ^19^, ^20^ 3D images based on Positron Emission Tomography/Computed Tomography (PET/CT) image fusion^21^, ^22^, ^23^, ^24^ or on Magnetic Resonance (MR),^25^ and molecular margin analysis^12^, ^26^ have been proposed to obtain HNSCC complete resection, but consensus has not been reached so far.^20^

Three-Dimensional tumor Segmentation (3DS) images obtained using a computer software (InVesalius software 3.0 version) have been reported as a viable alternative to complement the presurgical assessment of maxillary sinus cholesteatoma,^27^ ghost cell odontogenic carcinoma,^28^ and chordoma of the clivus.^29^ The 3DS model is a low-cost procedure, as the 3D images are obtained from the MPR images of CT scan or magnetic resonance, conventionally required at diagnosis to identify the tumor stage, and the InVesalius software 3.0 version is free of tax-exempt for use (https://www.cti.gov.br/invesalius).

We herein analyzed the role of 3DS images using InVesalius software 3.0 to define the locally advanced OSCC extension and found some encouraging results.

## Methods

**Figure 1:**
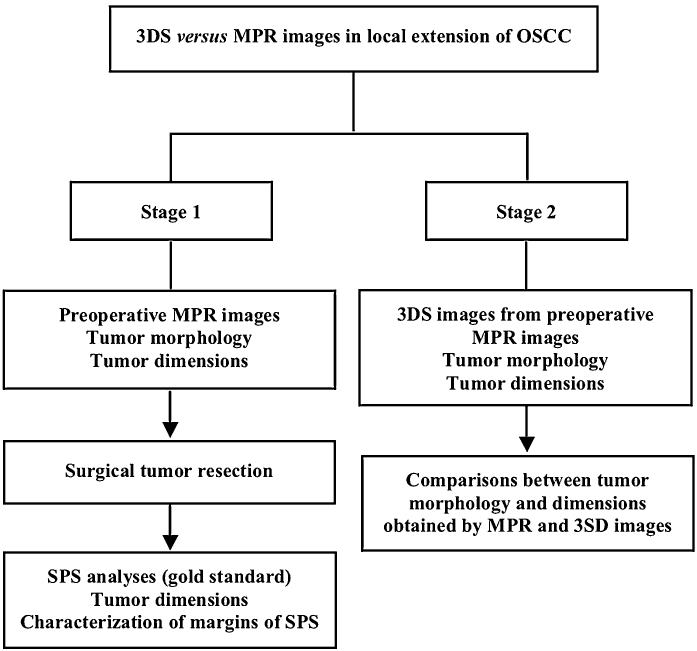
Flowchart of the study. In stage 1, Multiplanar Reconstruction (MPR) of computed tomography images were used to analyze morphology and dimensions of the Oral Squamous Cell Carcinoma (OSCC), and for planning of surgical tumor resection. After that, Surgical Pathology Specimens (SPS) were analyzed to obtain tumor dimensions and to characterize the margins as positives or negatives. In stage 2, Three-Dimensional Segmentation (3DS) images of preoperative MPR images were used to obtain tumor morphology and dimensions. Comparisons between tumor morphology and dimensions obtained by MPR and 3SD images were the last procedure of the study.

This study comprised patients with locally advanced OSCC submitted to surgery as the primary option of treatment. All patients were newly diagnosed cases, and had primary tumors considered clinically resettable with at least one centimeter of free margins, as previously recommended.^5^, ^7^ The study was conducted in two stages (Fig. 1).

**Figure 2:**
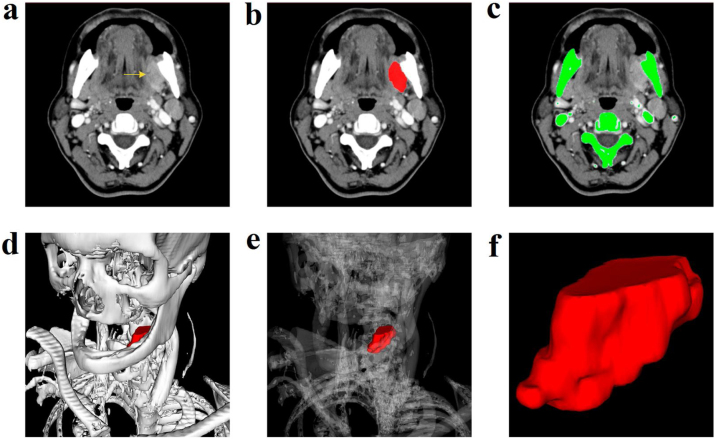
Contrast enhanced computed tomography scan: left retromolar trigone lesion (A). Manual segmentation of the tumor (B). Automatic segmentation of the skeleton (C). Tumor Three-Dimensional Segmentation (3DS) model along with modifiable bone transparency (D and E). Tumor spatial configuration (F).

In stage 1, patients were clinically staged (cTNM) by physical examination and contrast-enhanced CT (Fig. 2A) and thorax, according to the American Joint Committee on Cancer criteria (AJCC).^30^ All CT images for MPR were acquired in the same equipment without artifacts that could compromise the 3DS images (Aquilion 64 channels, Toshiba Medical System Corporation with multi-slice scanning system), and tumor morphology and axis were analyzed by the same specialist radiologist.

The surgical resection of the primary tumor and cervical lymph nodes was performed with curative intent in all patients using only MPR images without any sort of 3DS images. Intra-operative frozen sections were used to assist in clear margins.

The Surgical Pathology Specimens (SPS) were postoperatively evaluated by macroscopic and microscopic examination. All three dimensions of tumor were applied to the ellipsoid formula to determine its volume.^30^ The primary tumor measurements in the SPS were considered the gold standard for the study. The tumor pathological stage (pTNM) was defined based on SPS analyses, according to the American Joint Committee on Cancer criteria (AJCC).^31^

The 3DS images were obtained only in a second period of the study (stage 2) to meet the requirements of the local Ethics Committee. The 3DS images were obtained from MPR images using the InVesalius software 3.0 version, according to recommended procedures (https://www.cti.gov.br/invesalius). Segmentation and contouring of the tumors were performed manually (Fig. 2B). The images of the skeleton were performed automatically by selecting the proper thresholds (Fig. 2C) to obtain 3D images of the tumor along with bone structures (Fig. 2D). The computer software magnified and rotated images with bone transparency adjustment (Fig. 2E) to highlight tumor topography (Fig. 2F). Two researchers with unequivocal experience in the 3DS model and without knowledge of the SPS findings were responsible for obtaining and analyzing tumor morphology and dimensions.

Data comparisons were performed by summary statistics, Analysis of Variance (ANOVA) and Intraclass Correlation Coefficient (ICC). The level of significance was 95%. Tests were done using the SAS System for Windows (Statistical Analysis System), version 9.4 (SAS Institute Inc. 2002‒2008, Cary, NC, USA).

## Results

Twenty-six patients with locally advanced OSCC were enrolled in the current study. The mean and median ages of patients were 62 and 61 years, respectively. Most patients were male, and with a high percentage of alcohol and tobacco consumption. The most common primary location of the tumor was the tongue, and most patients had a moderately differentiated tumor. All patients had tumor at advanced stages (III or IV). SPS examination showed positive surgical margins in 11 out of 26 (42%) cases (Table 1).

**Table 1:** Clinicopathological aspects of 26 oral squamous cell carcinoma patients.

Variable	Number (SD, range or %)
**Age (years)**	
Average	62 ± 13
Median	61 (33 to 91)
**Gender**	
Male	22 (84.6)
Female	4 (15.4)
**Smoking**	
Yes	25 (96.0)
No	1 (4.0)
**Alcoholism**	
Yes	25 (96.0)
No	1 (4.0)
**Location of the tumor**	
Tongue	11 (42.3)
Floor of oral cavity	6 (23.1)
Lip	4 (15.4)
Retromolar trigone	4 (15.4)
Hard palate	1 (3.8)
**Tumor**’**s differentiation degree**	
Well differentiated	2 (7.7)
Moderately differentiated	22 (84.6)
Not classified	2 (7.7)
**Clinical stage (cTNM)**	
III	12 (46.2)
IV	14 (53.8)
**Muscle or bone or invasion**	
Present	21 (80.8)
Absent	5 (19.2)
**Pathological stage (pTNM)**	
III	6 (23.1)
IV	13 (50.0)
Not assessed	7 (26.9)
**Surgical margins**	
Free	15 (57.7)
Compromised	11 (42.3)

SD, Standard Deviation. Tumor clinical stage (cTNM) and pathological stage (pTNM) were established according to American Joint Committee on Cancer (AJCC) criteria. In two cases and in seven cases it was not possible to identify the tumor differentiation degree and pTNM, respectively, due to the lack of consistent information.

OSCC morphology and OSCC relationship to adjacent structures were more satisfactorily assessed by 3DS than MPR images through individual assessment of axial, coronal, and sagittal slices. Tumor dimensions were satisfactorily obtained by MPR images, 3DS images, and SPS analysis.

**Figure 3:**
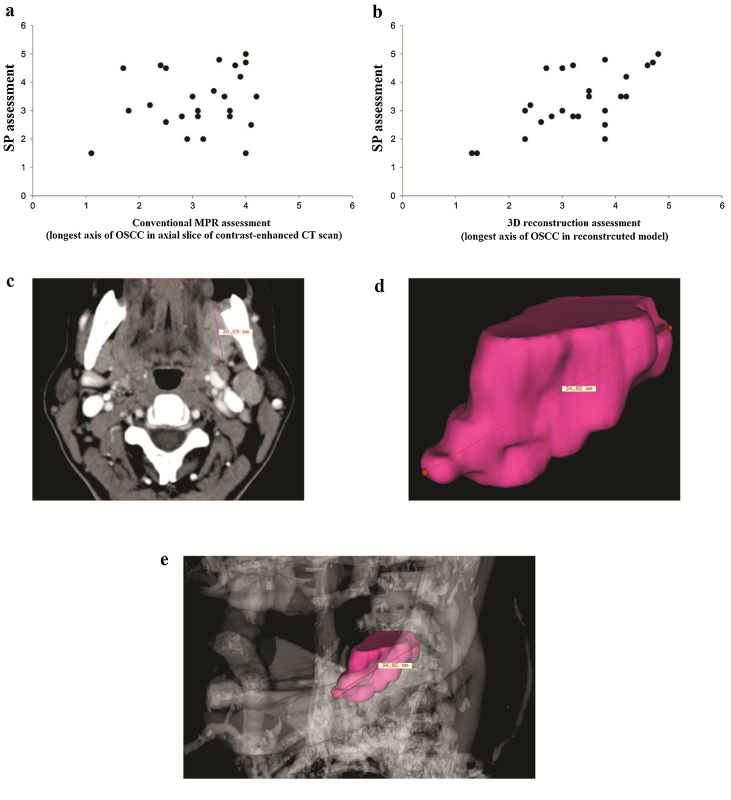
Dispersion graphics with moderate correlation between Multiplanar Reconstruction (MPR) images and Surgical Pathology Specimens (SPS) (A). Strong correlation between three-dimensional segmentation (3DS) model and SPS (B). Axial slice of MPR image (C) and 3DS image (D) of tumor measurements. 3DS image of tumor with skeleton (E).

Similar OSCC volume and longest axis was obtained by MPR images, SPS measurements and 3DS images (Table 2). A moderate correlation of longest axis was found between MPR images and SPS measurements (ICC = 0.51; 95% CI -0.09‒0.78) (Fig. 3A), whereas a strong correlation of tumor longest axis was found between 3DS images and SPS measurements (ICC = 0.82; 95% CI 0.59‒0.92) (Fig. 3B).

**Table 2:** Multiplanar reconstruction (MPR), three-dimensional segmentation (3DS) and surgical pathology specimen (SPS) assessments of 26 oral squamous cell carcinoma.

Variable	Mean	SD	Median	Min-Max	*P*-value
**Volume**					
MPR	8.11	6.38	7.20	0.57−24.19	0.29
3DS	7.29	4.40	4.76	0.45−17.14	
SPS	9.91	10.08	6.83	0.33−47.12	
**Longest axis**					
MPR	3.11	0.90	3.20	1.1−5.0	0.101
3DS	3.36	0.97	3.41	1.3−5.0	
SPS	3.47	1.20	3.35	1.5−6.5	

SD, standard deviation; Min, minimum; Max, maximum.

Considering only the SPS with positive margins, the MPR and 3D images underestimated the tumor’s longest axis in eight out of 11 (72.7%) cases and in 5 out of 11 (42.3%) cases, respectively.

A case of OSCC in which the tumor had its location, morphology and longest axis better assessed by 3DS than MPR images, with the SPS as the gold standard, is indicated in Fig. 3C‒3E.

## Discussion

In current practice, intraoperative assessment of the HNSCC free margin is dependent on visual appearance, palpation of the tumor, and intraoperative frozen sections for identification of residual tumor.^32^, ^33^ The rate of positive margins in these tumors has remained stagnant over the past three decades and is consistently associated with local recurrence and lower survival of OSCC patients.^6^ This suggests that significant improvements must be made during surgical planning and intraoperatively to ensure complete tumor resection. Several images that enable increasing the accuracy of the tumor longest axis and its boundaries prior or during the surgical procedure have been described to improve the quality of treatment of HNSCC patients.^16^, ^17^, ^18^, ^19^, ^20^, ^21^, ^22^, ^23^, ^24^, ^25^ In this pilot study, we aimed to verify whether 3DS images using the InVesalius software 3.0 version confers advantage over conventional MPR images in morphological characterization of OSCC as well as in measurements of its dimensions, using the SPS findings as a reference, and found some encouraging results.

We found that OSCC morphology and its relationship to adjacent structures were more accurately assessed by the 3DS than MPR images. This finding was not surprising, since the 3DS model was designed to amplify, rotate, and adjust bone transparency, thereby highlighting the topography and contours of tumors.^27^, ^28^, ^29^

We also found similar tumor volume and longest axis measured by MPR images, 3DS images and analysis of SPS. The similarity in tumor dimensions may reside in the fact that the 3DS images were obtained from MPR images and thus, differences between models would not be expected. On the other hand, the number of patients evaluated in the current study was relatively small and may not have been enough to show differences between the models. In this study, the strong correlation of the longest tumor axis found between 3DS images and SPS measurements while only a moderate correlation of longest axis was found between MPR images and SPS measurements suggest that 3DS model is superior to MPR for this determination and support our second hypothesis. Moreover, we found positive margins in 42.3% of SPS, as previously reported,^32^, ^33^, ^34^ and when only SPS with positive margins were taking into account, 3D images underestimated the tumor’s longest axis in lower number of cases than MPR images.

A 3D-navigation system based on PET/CT image fusion was seen as a useful tool to assess and improve local control in advanced HNSCC in the study carried out by Feichtinger et al.^21^ Ibraginov et al.^25^ described a segmentation of tongue muscles from high-resolution MR images combined with whole tongue segmentation from dynamic low-resolution MR images as an important method for oral cancer surgery planning. Zrnc et al.^22^ analyzed a small number of HNSCC patients who underwent surgical treatment with 18-FDG PET/CT image-fusion using a 3D navigation-system based workstation, obtained image-guided needle biopsies within the tumor, and observed that PET scans may overestimate tumor extension. 3D FDG PET segmentation images of 47 HNSCC were analyzed by Smith et al.;^23^ the authors concluded that the model is useful in analysis of tumor extension, but reproducibility of data requires reduction of errors owing to segmentation methods. Debacker et al.^24^ used high-resolution^18^ FDG PET/CT to better assess the status of 3D intraoperative margins, and proposed further optimization and patient stratification to improve the clinical implementation of the method. In summary, the results found in the current study and in previous studies suggest that 3D images may improve HNSCC characterization and complete resection, but larger studies are still required to confirm its use in clinical practice.

Other new technologies have been proposed to improve the assessment of HNSCC morphology and limits. Keereweer et al.^16^ described a NIR fluorescence optical imaging during surgery as having the potential of to identify malignant lesions before becoming visible to the naked eye. NIR fluorescence optical imaging-guided surgery showed improved HNSCC resection quality in animal models in studies conducted by Atallah et al.^17^ and Christensen et al.^19^ Iqbal & Pan^18^ postulated in a revision article that NIR guided fluorescent surgery allowed the detection of residual microscopic disease in lymph nodes and other tissues. Moreover, molecular margin analysis has been indicated to distinguish normal from pathological tissues; intraoperatively, this information may be used to guide resection, while postoperatively, it may help to stratify patients for adjuvant treatment.^26^ Although these methods seem to be more effective to identify residual tumors,^12^, ^20^ their cost and complexity may limit their use to large-scale services.

## Conclusion

Despite the small sample size and limited statistical analyses, our data suggest that the 3DS model represents a useful tool for surgical planning complete OSCC resection, but confirmation in a larger cohort of patients is required. If so, it is possible that the low-cost 3DS model can be used to complement palpation, visual inspection, and frozen sections for the identification of residual tumor in OSCC resection in cancer patient care services of different levels of complexity and variable financial resources.

## Ethics in publishing

This study was approved by the local Ethical Committee (number: 970.160). All procedures were carried out according to the Helsinki Declaration, and informed consent was obtained.

## Funding

The study was conducted with financial support of Coordenação de Aperfeiçoamento de Pessoal de Nivel Superior (CAPES).

## Conflicts of interest

The authors declare no conflicts of interest.
